# Multiple Level CT Radiomics Features Preoperatively Predict Lymph Node Metastasis in Esophageal Cancer: A Multicentre Retrospective Study

**DOI:** 10.3389/fonc.2019.01548

**Published:** 2020-01-21

**Authors:** Lei Wu, Xiaojun Yang, Wuteng Cao, Ke Zhao, Wenli Li, Weitao Ye, Xin Chen, Zhiyang Zhou, Zaiyi Liu, Changhong Liang

**Affiliations:** ^1^School of Medicine, South China University of Technology, Guangzhou, China; ^2^Department of Radiology, Guangdong Provincial People's Hospital, Guangdong Academy of Medical Sciences, Guangzhou, China; ^3^Department of Radiology, The Sixth Affiliated Hospital, Sun Yat-sen University, Guangzhou, China

**Keywords:** esophageal squamous cell carcinoma, lymph node metastasis, radiomics, computer vision, deep learning

## Abstract

**Background:** Lymph node (LN) metastasis is the most important prognostic factor in esophageal squamous cell carcinoma (ESCC). Traditional clinical factor and existing methods based on CT images are insufficiently effective in diagnosing LN metastasis. A more efficient method to predict LN status based on CT image is needed.

**Methods:** In this multicenter retrospective study, 411 patients with pathologically confirmed ESCC were registered from two hospitals. Quantitative image features including handcrafted-, computer vision-(CV-), and deep-features were extracted from preoperative arterial phase CT images for each patient. A handcrafted-, CV-, and deep-radiomics signature were built, respectively. Then, multiple radiomics models were constructed by merging independent clinical risk factor into radiomics signatures. The performance of models were evaluated with respect to the discrimination, calibration, and clinical usefulness. Finally, an independent external validation cohort was used to validate the model's predictive performance.

**Results:** Five, seven, and nine features were selected for building handcrafted-, CV-, and deep-radiomics signatures from extracted features, respectively. Those signatures were statistically significant different between LN-positive and LN-negative patients in all cohorts (*p* < 0.001). The developed multiple level CT radiomics model that integrates multiple radiomics signatures with clinical risk factor, was superior to traditional clinical factors and the results reported by existing methods, and achieved satisfactory discrimination performance with C-statistic of 0.875 in development cohort, 0.874 in internal validation cohort and 0.840 in independent external validation cohort. Nomogram and decision curve analysis (DCA) further confirmed our method may serve as an effective tool for clinicians to evaluate the risk of LN metastasis in patients with ESCC and further choose treatment strategy.

**Conclusions:** The proposed multiple level CT radiomics model which integrate multiple level radiomics features into clinical risk factor can be used for preoperative predicting LN metastasis of patients with ESCC.

## Introduction

Esophageal cancer (EC) is the seventh most common cancer worldwide and the sixth leading cause of cancer death overall, with an estimated 572,000 new cases and 509,000 deaths in 2018 (1). Esophageal squamous cell carcinoma (ESCC) is the major histological subtype of EC, especially in high-incidence areas such as China (2, 3). EC is often associated with a poor prognosis, and the 5-year relative survival rate during 2008 through 2014 was 19% (4). Lymph node (LN) metastasis is one of the most important prognostic factor, which generally indicates a worse outcome (5). Accurate preoperative LN staging is also important for making treatment decisions, such as neoadjuvant chemoradiotherapy (6). Therefore, assessing LN status preoperatively in patients with EC is of clinical importance.

Currently, computed tomography (CT) plays an important role in preoperative nodal staging in patients with EC. However, its ability in identifying positive LN is unsatisfactory, and the reported accuracy, sensitivity, and specificity are 54.5, 39.7, and 77.3%, respectively (7). The low accuracy may result in patients being under- or over-staged. Clinical determination of LN metastasis according to LN size criteria on preoperative CT is limited. Recently, radiomics, as an emerging tool, has shown potential values in predicting LN metastasis by extracting high-throughput quantitative features from medical images (8–10). However, most of the features extracted are defined by mathematical formulas (also called handcrafted feature), which are shallow, susceptible to noise, and low-order image features. These features may not be sufficient to reveal tumor heterogeneity and to predict LN metastasis in patients with ESCC (11).

To overcome these limitations, several new strategies, such as computer vision and deep learning have been proposed. On one hand, computer vision features (CVFs), including local and global features, are being applied widely in traditional image processing (12–14). Compared to handcrafted features, CVFs have the advantages of rotation invariant, insensitive to noise. These advantages have the potential to avoid the effects of noise that affecting handcrafted features on CVFs. Several studies have used CVFs to achieve disease diagnosis and prognosis prediction in medical imaging (15, 16).

On the other hand, deep learning has drawn increased interest, among which convolutional neural network (CNN) shows great image classification and recognition performance in medical imaging in recent years (17, 18). Compared to handcrafted radiomics features, the deep features are extracted from pixel images directly and reflect tumor information from a different perspective, which may add predictive value for prediction of LN status in patients with ESCC (11). Although the medical image dataset is typically not sufficient for deep learning which requires millions of weights to learn, the transfer learning is proposed to cover the shortage. Transfer learning, which uses pre-trained models from images of other domains and makes these useful for a new dataset (19), is currently widely used in the deep learning medical field (20).

Several studies have shown substantially predictive value improvement of the multiscale model that integrating multiple signatures compared to the use of individual signature (21, 22). We hypothesized that multiple level radiomics model have potential value in preoperative prediction of LN metastasis in patients with ESCC. Therefore, the aim of the current study was to develop a multiple level CT radiomics model, which integrated handcrafted-, CV-, and deep-radiomics signatures, to improve the performance of the LN metastasis prediction in patients with ESCC, and validate it within an independent external dataset.

## Materials and Methods

### Ethics Statement

This multicenter retrospective study was approved by the Institutional Ethics Committee of two participating hospitals (Guangdong Provincial People's hospital, denote as Hospital 1; The Sixth Affiliated Hospital, Sun Yat-sen University, denote as Hospital 2). Requirement for informed consent was waived.

### Study Population

**Figure 1:**
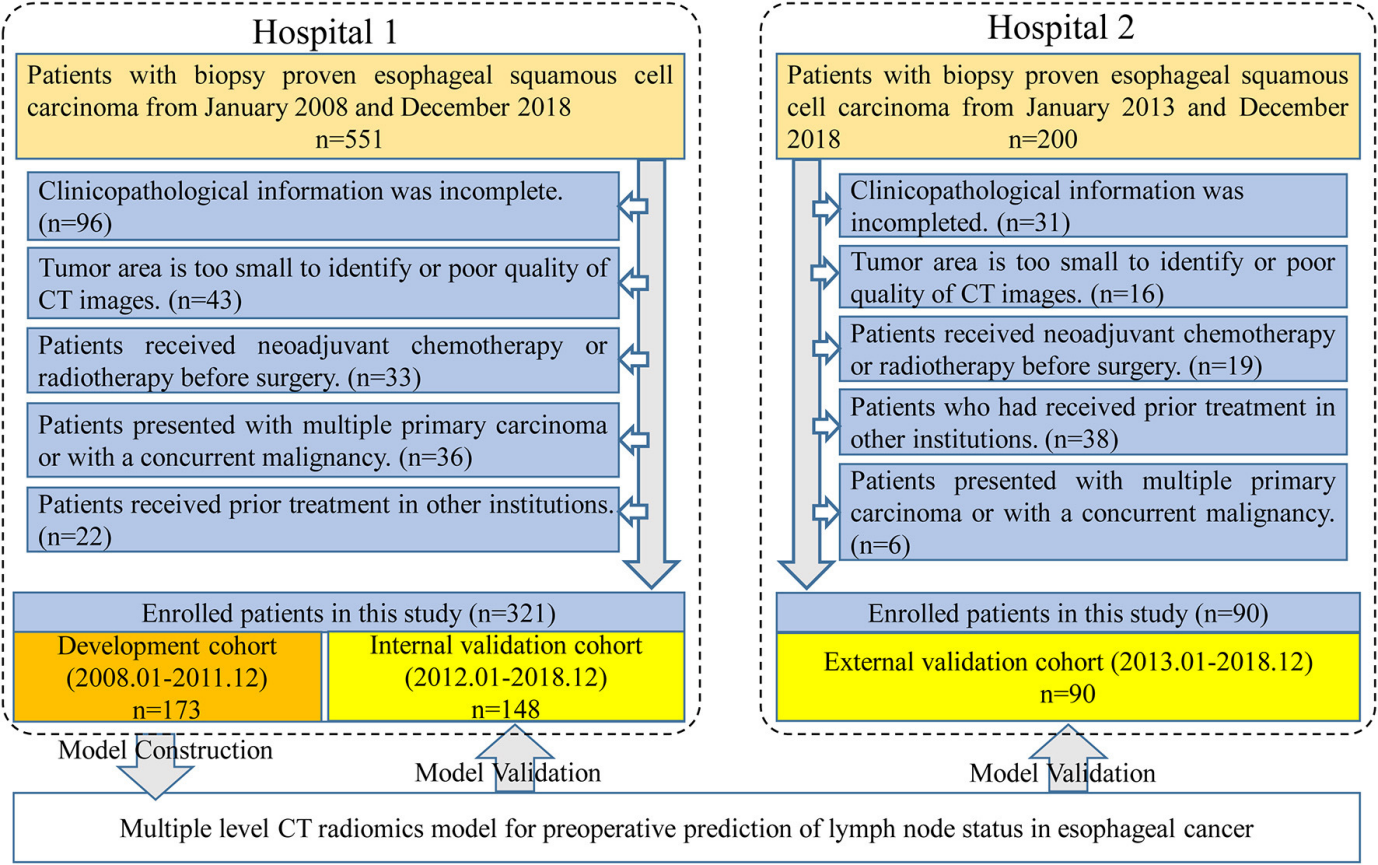
Data screening flowchart and study design. In total, 751 patients were collected from two hospitals but only 411 patients met our research requirements. One hundred and seventy-three patients in Hospital 1 were used for model training and the others in Hospital 1 were used for internal validation. Ninety patients from Hospital 2 were used as an independent external validation.

Four hundred and eleven patients were enrolled from two hospitals (Hospital 1: *n* = 321, Hospital 2: *n* = 90) in this study. Our inclusion criteria were as follows: (a) patients with histologically confirmed ESCC; (b) patients who underwent standard contrast-enhanced CT examination within 2 weeks before surgery; (c) patients who received radical esophagostomy with extensive lymph node dissection; (d) patients who had pathologically confirmed LN status after surgery. Exclusion criteria included: (a) patients who received preoperative neoadjuvant chemotherapy or radiotherapy; (b) patients who had received prior treatment in other institutions; (c) patients who presented with multiple primary carcinoma or with a concurrent malignancy; (d) patients whose tumor lesion was too small to identify or had poor quality of CT images; (e) clinicopathological information was incomplete. A more detailed description of the data is presented in the Figure 1. Three hundred twenty-one patients from Hospital 1 were chronologically divided into two cohorts: the development cohort with 173 patients who were treated between January 2008 and December 2016, and the internal validation cohort with 148 patients who were treated between January 2017 and December 2018. An external validation cohort with 90 patients between January 2017 and December 2018 from Hospital 2 was used for independent validation.

Baseline clinical and histopathological information of the enrolled patients were derived from the clinical records and pathology reports. Tumor location was determined according to the 8th edition of the American Joint Committee on Cancer (AJCC) Cancer Staging Manual (23). Histologic grade was obtained from pathology reports. CT-reported LN status was estimated on the preoperative CT images by a radiologist who with 12 years of experience in upper gastrointestinal CT interpretation. A positive lymph node was defined as the short axis diameter of the largest regional LN >10 mm (24). Besides, the age and gender were also obtained for each patient.

### Images Acquisition and Processing

All patients have underwent a contrast-enhanced CT scans from the neck to the abdomen. Scan parameters are listed in the Supplementary Dataset. Images were reconstructed with a slice thickness of 5 mm in Hospital 1 and 1 or 1.5 mm slice thickness in Hospital 2.

For handcrafted features, CVFs and deep features extraction, a region of interest (ROI) was outlined along the tumor border with exclusion of the necrosis and air area in the largest cross-sectional area of the CT images using a free software called ITK-SNAP (version 3.6.0, http://www.itksnap.org). To evaluate the reproducibility of the extracted features, we randomly selected 50 samples from the development cohort to extract features and analyze the repeatability with inter- and intra-class correlation coefficients (ICC) indicators. Normally, features with ICC > 0.75 were defined as good agreement in reproducibility (25). The ROI delineation was performed by two radiologists, Reader 1 and Reader 2, with 12 and 15 years of upper gastrointestinal CT interpretation experience, respectively.

### Multiple Level Radiomics Features Extraction

#### Handcrafted Radiomics Features Extraction

**Figure 2:**
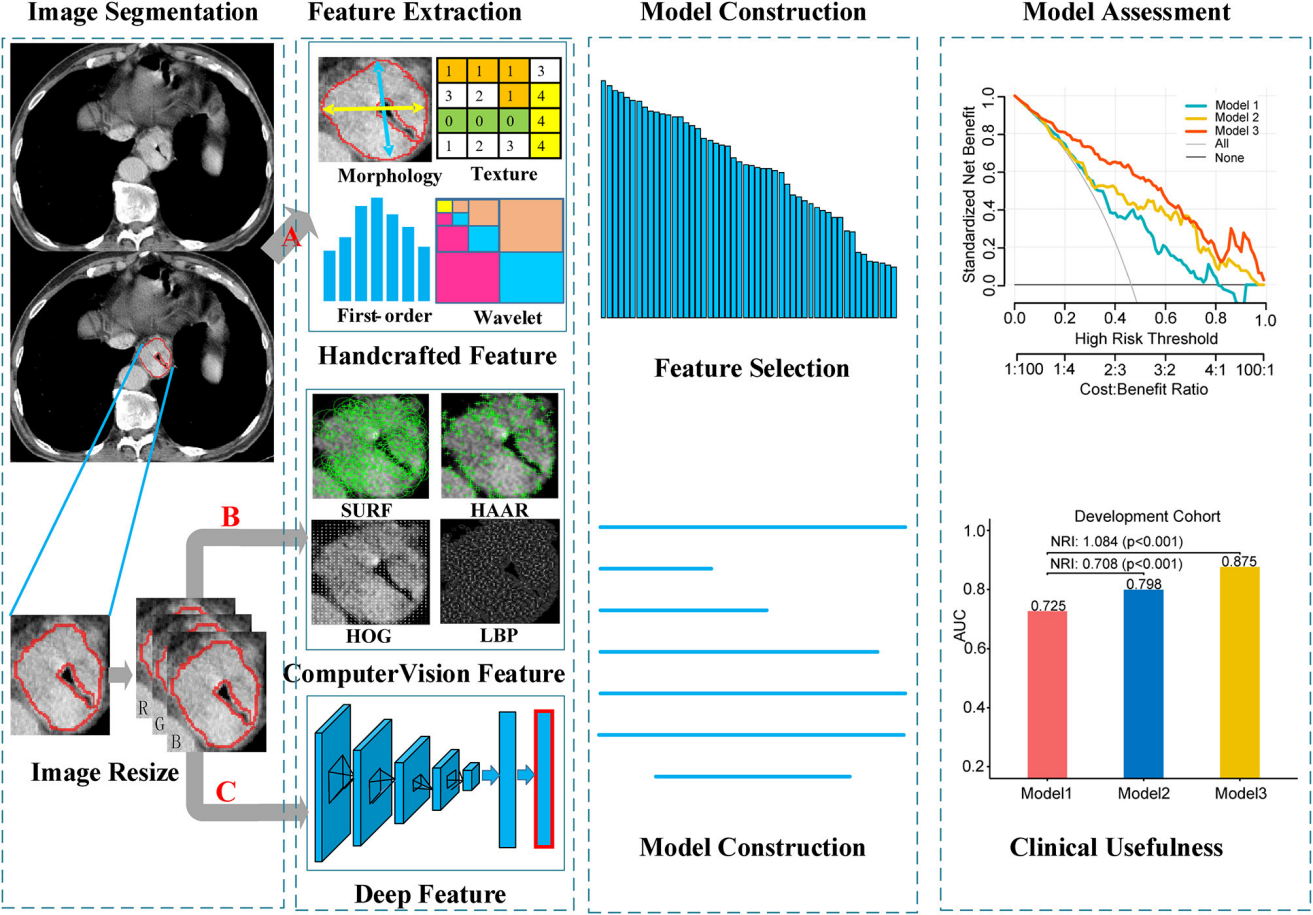
Workflow of the radiomics model building process. Image segmentation was performed by experienced radiology doctor on the CT image. The handcrafted features were extracted from the segmented image. For computer vision features and deep features, sub-images contain whole tumor were clipped from the segmented images, and then combined into a RGB image. Computer vision features and deep features were extracted from the RGB images. **(A)** Segmented images for extracting handcrafted features. **(B,C)** RGB images for computer vision and deep features extraction, respectively.

The image data analyzed in this study were derived from various CT scanners. In order to reduce the impact of machine factors, all images had been normalized before feature extraction. A toolbox of radiomics feature extraction based on the Matlab 2016b was developed in-house. All images were normalized by a min-max normalization algorithm with the Hounsfield units transformed into a range of [1, 100]. Then, four types of handcrafted radiomics features were extracted for further analysis: (a) 14 quantitative features described the size of tumor, called first-order statistics features, (b) 7 quantitative features described the tumor intensity, called size- and shape-based features, (c) 63 texture features reflected the intratumoral heterogeneity, and (d) 3,388 features were derived from wavelet filter and Laplace-Gaussian filter. A total of 3,472 handcrafted radiomics features were extracted in each patient (Figure 2). More detailed description about the handcrafted features were presented in the Methods S1.

#### Local Features Based on Computer Vision Extraction

Local features (also called local descriptors), which are distinctive and invariant to intensity variation, noise and distortion, have been widely utilized in computer vision filed and digital image processing. In this study, local features based on CV were extracted from the segmented images, which could be categorized as four types: (a) Local Binary Pattern (LBP); (b) Histogram of Oriented Gradients (HOG); (c) Speeded Up Robust Features (SURF); (d) Haar-like features. In total, 5,126 CVFs were computed based on Python 3.5 (https://www.python.org/) in this article (Figure 2). Regarding the machine vision features, we provided a detailed description in the Methods S2.

#### Deep Radiomics Features Extraction

Deep feature extraction was executed with Matlab 2016b using a toolbox called MatConvNet (version 1.0-beta25; http://www.vlfeat.org/matconvnet/). Convolution Neural Network-Fast (CNN-F), a pre-trained CNN model was selected to extract the deep features. In this paper, deep features were generated from pre-trained CNN-F models through transfer learning.

CNN-F contains eight learnable layers, five of which are convolutional layers, and the last three are fully connected layers. This model was pre-trained on ImageNet Large Scale Visual Recognition Challenge 2012 (ILSVRC-2012) dataset and the input was a fixed-size 224 × 224 pixel^2^ RGB images. In order to match the input of the pre-trained CNN-F model, three steps were performed for each patient. First, the largest tumor area slicer was selected from all slicers for each patient, and manually segmented the tumor area along the tumor boundary. Then, cropped the segmented tumor area and resized to 224 × 224 pixel^2^ by bicubic interpolation. Finally, the resized single channel image was encoded into a three-channel image and allowed to input the model. When deep feature extraction was performed, the last fully connected layer was removed, and only the information of the seventh fully-connected layer was extracted as the deep feature and used for subsequent analysis (Figure 2). The hyperparameters of the model were the same as that used by (26): momentum 0.9, weight decay 5 × 10^−4^, initial learning rate 10^−2^. When the validation error stopped decreasing, the initial rate dropped to one tenth. Other relevant descriptions about the deep features are presented in the Methods S3.

### Feature Selection

In order to select effective features for prediction signatures construction, a coarse to fine feature selection strategy was adopted. Firstly, to ensure reproducibility of features, a subset cohort was randomly extracted from development cohort as mention above. ICCs was used to assess the reproducibility of features. Normally, features with ICCs above 0.75 were considered high agreement in reproducibility. Secondly, the correlation coefficient value for all pairs of features were calculated. All pairs of features with correlation coefficient over 0.9 were detected, and the features in each of those pairs with the high predictive (AUC value decide) were retained. Thirdly, Random Forest-Recursive Feature Elimination (RF-RFE) algorithm was applied. RF-RFE is an automatic method for feature selection, which begins by fitting a model on the entire set of features and calculating an importance score for each feature, and then removing the less relevant features. This process iterates over and over until the optimal feature set is selected. Finally, backward stepwise regression was used to select key features for LN metastasis prediction.

The feature selection strategy was applied to the handcrafted, CV and deep radiomics feature selection process. In order to maintain the independence between the development and the validation cohort, feature selection was only performed on the development cohort, and validation cohort was only used to evaluate the prediction performance of the model.

### Signatures Building and Model Development

After feature selection, radiomics signature was built in the development cohort with selected key features by using logistic regression for handcrafted, CV and deep learning, respectively. Meantime, radiomics scores could be calculated for each patient. The association between signatures and LN metastasis were assessed in each cohort.

To assess the efficacy of radiomics signatures in predicting LN metastasis of patients with ESCC compared to prior studies, we constructed three models. First, based on prior studies (27), a model (called Model 1) consisting of clinical indicator and handcrafted radiomics features was constructed. Then, CV radiomics signature was integrated into Model 1 to form the Model 2. Finally, the deep radiomics signature was merged into the Model 2 to form the Model 3 (**Table 2**).

### Models Performance Assessment

To assess the performance of prediction models, four steps recommended by Steyerberg et al. (28) were applied in this study:

Step 1: model overall performance
  Brier score (29) and Nergekerke's *R*^2^ (30) were applied to assess the overall performance for all models in this study. The Brier score provided a measure of the agreement between the observed binary outcome (i.e., LN positive vs. LN negative in this study) and the predicted probability of that outcome. The brier score was computed as $\sum {\left(y_{j}-prob_{j}\right)}^{2}/N$, with *y* the outcome and *prob* the predicted probability for sample *j* in the data set of *N* samples. Brier score ranges from 0 for a perfect prediction model to 0.25 for useless prediction model. The Nergekerke's *R*^2^ was a measure of explained variation computed on the log-likelihood scale.
Step 2: model discrimination
  The discriminative ability of model was evaluated using concordance statistic (*C*-statistic) and discrimination slope. *C*-statistic, in binary outcome, is equivalent to the area under the receiver operating characteristic curve. A reasonable discrimination is signaled by the *C*-statistic values of 0.7–0.8 and a good discrimination by values surpassing 0.8 (31). Discrimination slope is defined as the slope of a linear regression of predicted probabilities of events derived from a model on the binary event status, which reflects the models how well samples with and without the outcome are separated. Discrimination box plot can more intuitively reflect the discrimination ability of the model, which will show less overlap between those with and without the outcome for a better discriminating model.
  Net Reclassification Improvement (NRI) is a statistic that measures the incremental prognostic values that a new marker will improve when added to an existing prediction model, which offers a simple and intuitive way to quantify the improvement ability of marker.
Step 3: model calibration
  Calibration refers to how closely the predicted probabilities of LN metastasis agree with the observed LN metastasis in this study. The calibration curve could provide an intuitive representation of the consistency between predicted and observed outcome. Perfect prediction should be corresponding to 45° line. Calibration slope was measured to reflect the average strength of the predictor effects. The Hosmer–Lemeshow test was also applied to check the goodness-of-fit of the model. A reasonable calibration should have a higher *p*-value (>0.05).
Step 4: model clinical usefulnes
  In addition to assessing the discrimination and calibration of the models, we also hoped to know whether the prediction model was beneficial in clinical practice. Therefore, we also evaluated the clinical usefulness of the models using decision curve analysis (DCA). Standardized net benefit (sNB) was conducted derived from decision curve.

Standardized net benefit was conducted as a function of the risk threshold derived from decision curve (sNB value ranges from 0 to 1). Once the threshold was applied to grouped patients into low risk and high risk, sensitivity, and specificity were often calculated, and used as measures for usefulness. The clinical impact plot and ROC components plot were also conducted for assessing the clinical usefulness of models.

### Statistical Analysis

All statistical analyses were performed using the R programming language (version 3.4.2; https://www.r-project.org/). The R packages used in this study were listed in the Methods S5. All statistical tests in this study were two-sided and considered statistically significant if *p* ≤ 0.05. Chi-square test was applied for categorical variables, such as sex, tumor location, histologic grade, and CT-reported LN status. Continuous variables such as age, and radiomics score were analyzed using the Mann–Whitney *U*-test.

## Results

### Clinical Characteristics

As displayed in Figure 1, a total of 751 entitle patients were consecutively registered in this study from the two hospitals, and 340 patients were excluded through the exclusion criteria. Finally, 411 patients were registered for further analysis. The dataset from Hospital 1 was chronologically divided into the development cohort and internal validation cohort, the dataset from Hospital 2 were used as external validation cohort. The clinical characteristics of all patients were shown in Table 1.

**Table 1:** Characteristics of patients with ESCC in development and validation cohorts.

	**Development cohort** (***n*** **= 173)**			**Internal validation cohort (*****n*** **= 148)**			**External validation cohort (*****n*** **= 90)**		
**Characteristic**	**LNM (–)** **(*n* = 93)**	**LNM (+)** **(*n* = 80)**	***p***	**LNM (–)** **(*n* = 77)**	**LNM (+)** **(*n* = 71)**	***p***	**LNM (–)** **(*n* = 50)**	**LNM (+)** **(*n* = 40)**	***p***
**Age (mean ± SD, year)**	57.83 ± 8.51	56.74 ± 8.09	0.389	58.91 ± 8.09	57.63 ± 8.15	0.342	59.86 ± 8.48	58.65 ± 9.58	0.533
**Sex, No. (%)**			0.445			0.695			0.507
Male	70 (75.27)	65 (81.25)		61 (79.22)	59 (83.10)		40 (80.00)	35 (87.50)	
Female	23 (24.73)	15 (18.75)		16 (20.78)	12 (16.90)		10 (20.00)	5 (12.50)	
**Tumor location (%)**			0.135			0.082			0.362
Up	11 (11.83)	7 (8.75)		14 (18.18)	5 (7.04)		8 (16.00)	3 (7.50)	
Medium	45 (48.39)	29 (36.25)		32 (41.56)	28 (39.44)		25 (50.00)	19 (47.50)	
Low	37 (39.78)	44 (55.0)		31 (40.26)	38 (53.52)		17 (34.00)	18 (45.00)	
**Histologic grade (%)**			0.438			0.130			0.945
Well differentiated	18 (19.36)	10 (12.5)		12 (15.58)	11 (15.49)		11 (22.00)	8 (20.00)	
Moderately differentiated	48 (51.61)	47 (58.75)		48 (62.34)	34 (47.89)		27 (54.00)	23 (57.50)	
Poorly differentiated	27 (29.03)	23 (28.75)		17 (20.08)	26 (36.62)		12 (24.00)	9 (22.50)	
**CT-reported LN status (%)**			<0.001			<0.001			0.007
LN-negative	61 (65.59)	29 (36.25)		52 (67.53)	24 (33.80)		33 (66.00)	14 (35.00)	
LN-positive	32 (34.41)	51 (36.75)		25 (32.47)	47 (66.20)		17 (34.00)	26 (65.00)	

*LNM, lymph node metastasis; LN, lymph node; CT, computed tomography*.

The LN metastasis positives rate in the development, internal validation and external validation cohorts were 46.2, 47.9, and 44.4%, respectively. There was no significant difference between two groups with regard to age, gender, tumor location, and histological grade in three cohorts (*p*: 0.082–0.945).

### Feature Selection, Signature Construction, and Assessment

In total, 3,472 handcrafted, 5,126 computer vision, and 4,096 deep features were extracted for each patient. With the coarse to fine feature selection strategy, five, seven, and nine features were finally selected from the handcrafted features, CVFs, and deep features, respectively.

A handcrafted radiomics signature was built with a logistic regression using the five selected handcrafted features. The computer vision radiomics signature and deep radiomics signature were built with seven and nine features in the same way. Radiomics score in each cohort was also computed (Methods S4). In the development and validation cohorts, three signatures showed statistically significant differences between LN-positive and LN-negative patients (all *p* < 0.001, shown in Table S1).

### Model Development and Overall Assessment

For univariate analysis, CT-reported LN status, a clinical factor, was found significantly associated with LN status (*p* < 0.001, shown in Table 1). Thus, we built a model (called Model 1) using the CT-reported LN status and handcrafted radiomics signature by a logistic regression. Then, to evaluate the improved performance of CV radiomics signature, the computer vision CV radiomics signature was added into the Model 1 to form Model 2. Similarly, to facilitate the assessment of multiple level CT radiomics potential value, CV radiomics signature and deep radiomics signature were merged into Model 1 to develop Model 3 (Table 2).

**Table 2:** Risk factors for lymph node metastasis in patients with ESCC.

**Intercept and variables**	**Model 1**			**Model 2**			**Model 3**		
	**β**	**OR (95% CI)**	***p***	**β**	**OR (95% CI)**	***p***	**β**	**OR (95% CI)**	***p***
Intercept	0.172		0.333	0.310		0.109	0.474		0.036
CT-reported LN status	1.039	2.826 (1.657 4.917)	0.0002	0.907	2.476 (1.395 4.467)	0.002	1.011	2.748 (1.437 5.427)	0.003
Handcrafted-radiomics signature	1.051	2.860 (1.582 5.644)	0.001	1.190	3.286 (1.705 7.020)	<0.001	0.791	2.205 (1.116 4.858)	0.036
Computer vision-radiomics signature	–	–	–	0.997	2.710 (1.762 4.386)	<0.001	1.012	2.752 (1.706 4.679)	<0.001
Deep-radiomics signature	–	–	–	–	–	–	0.967	2.629 (1.820 4.040)	<0.001

*β, regression coefficient; OR, odds ratio; CI, confidence interval*.

Model 3 was the best model for LN status prediction in patients with ESCC, with good discrimination achieved (C-statistic, 0.875, 0.874, and 0.840 in development, internal validation and external validation cohort, respectively) (Table 3). Compared with Model 1, the overall performance of clinical predictor combining both handcrafted- and CV-radiomics signatures was improved: Nagelkerke's *R* increased from 20.6 to 37.1% and decreased from 20.9 to 17.6% for brier score (Table 3). Also, the discriminative capability was improved to 0.798, 0.27 for C-statistic and discrimination slope, respectively. Moreover, the sNB also was rose from 0.363 to 0.412 by adding the CV radiomics signature.

**Table 3:** Performance measures of ESCC LN metastasis prediction models in development and validation cohorts.

	**Development cohort**			**Internal validation cohort**			**External validation cohort**		
**Performance measures**	**Model 1**	**Model 2**	**Model 3**	**Model 1**	**Model 2**	**Model 3**	**Model 1**	**Model 2**	**Model 3**
**Overall**									
Brier	0.209	0.176	0.146	0.205	0.188	0.155	0.208	0.188	0.173
*R*^2^	0.206	0.371	0.513	0.243	0.328	0.484	0.213	0.322	0.406
**Discrimination**									
C-statistic	0.725	0.798	0.875	0.746	0.799	0.874	0.728	0.791	0.840
Discrimination slope	0.157	0.270	0.424	0.173	0.278	0.417	0.169	0.320	0.403
**Calibration**									
Calibration slope	1	1	1	1.083	0.860	0.956	0.951	0.854	0.803
H-L test (*p*-value)	0.301	0.544	0.692	0.504	0.793	0.420	0.186	0.411	0.063
**Clinical usefulness (T**_**50%**_**)**									
sNB(0.5)	0.363	0.412	0.562	0.296	0.394	0.606	0.275	0.375	0.450
Accuracy	0.705	0.728	0.798	0.662	0.703	0.791	0.689	0.722	0.711

*H-L test, Hosmer-Lemeshow test; sNB(0.5), standardized net benefit, threshold 0.5*.

Similarly, after adding the deep radiomics signature into the Model 2 to form Model 3, the Model 3 has been significantly improved in the discriminative ability, whether compared to the Model 1 or the Model 2 (Table 3).

In clinical usefulness, DCA was adopted for evaluating CV- and deep- radiomics signature based models for predicting LN status. A risk threshold of 0.5 was selected, which implied a relative weight of 1:1 between true-positive decisions and false-positive decisions. At point of 0.5, the sNBs of Model 1, 2, and 3 are gradually improved, which were 0.363, 0.412, and 0.562 in development cohort, respectively (**Figure 4**, Table 3).

### Model Performance Validation in Internal and External Cohort

**Figure 3:**
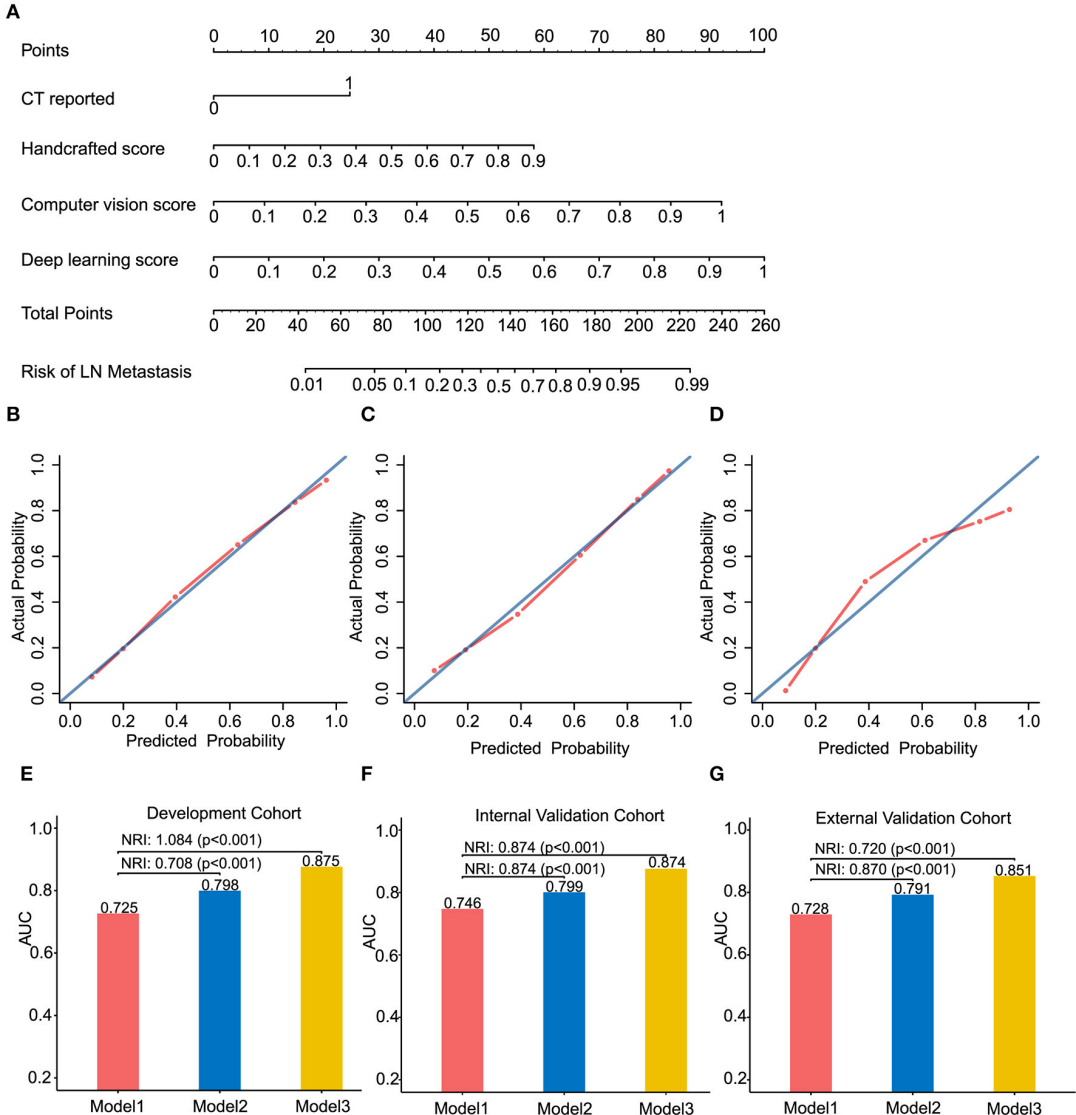
Radiomics nomogram of Model 3 for predicting the ESCC patients with LN metastasis **(A)**. Calibration curves of the radiomics nomogram in development cohort **(B)**, internal validation cohort **(C)** and external validation cohort **(D)**. Calibration curves reflect the calibration of Model 3 in terms of agreement between the predicted of LN metastasis and observed of LN metastasis. The 45-degree blue diagonal line represents a perfect ideal model. The closer the red dot-dash line is to the diagonal line, the better the prediction. **(E–G)** presents AUC values on the development, internal validation, and external validation cohort of Model 1, 2, and 3. Potential incremental value of models 2 and 3 relative to model 1 were evaluated by net reclassification improvement (NRI). **(B,E)** for development cohort, **(C,F)** for internal validation cohort, and **(D,G)** for external validation cohort.

The overall model performance in the external validation cohort with 90 patients (40 with LN metastasis) was lower than in the development and internal cohort. As an illustration, Model 3 decreased in *R*^2^ (0.406 instead of 0.484 and 0.513 in the development and internal validation cohort, respectively), but slightly increased in brier score (0.173 instead of 0.155 and 0.146 in the development and internal validation cohort, respectively). In terms of the discrimination ability, compared with the development and internal validation cohort, the C-statistic demonstrated a slight decrease in external validation cohort, but it was still the most discriminative model with high classification accuracy model (C-statistic above the 0.84 for Model 3, but Model 1 and 2 are below 0.8, in all cohorts). This could also be explained from the discrimination slope (Figure S1) of the models. Calibration curves of models in all cohorts were shown in Figures 3B–D. Calibration slope range from 0.803 to 1.083, and the Hosmer-Lemeshow test was of no statistical significance (*p* > 0.05). At the risk threshold of 0.5, the sNBs were better than other models in Model 3 (i.e., 0.450 > 0.375 > 0.275, in external validation).

### Assessing the Incremental Predictive Ability of the Models

We assessed the improvement of model performance introduced by inclusion of CV- and deep-radiomics signature based on the Model 1. The increase in the AUC showed statistic differences between Model 1 and Model 2 (Delong test: *p* < 0.001). NRI was also calculated and presented in Figures 3E–G. Likewise, with the addition of CV- and deep-radiomics signature, the reclassification ability of Model 3 was significantly improved compared Model 1. Detail results were showed in Table S2.

### Clinical Usefulness

**Figure 4:**
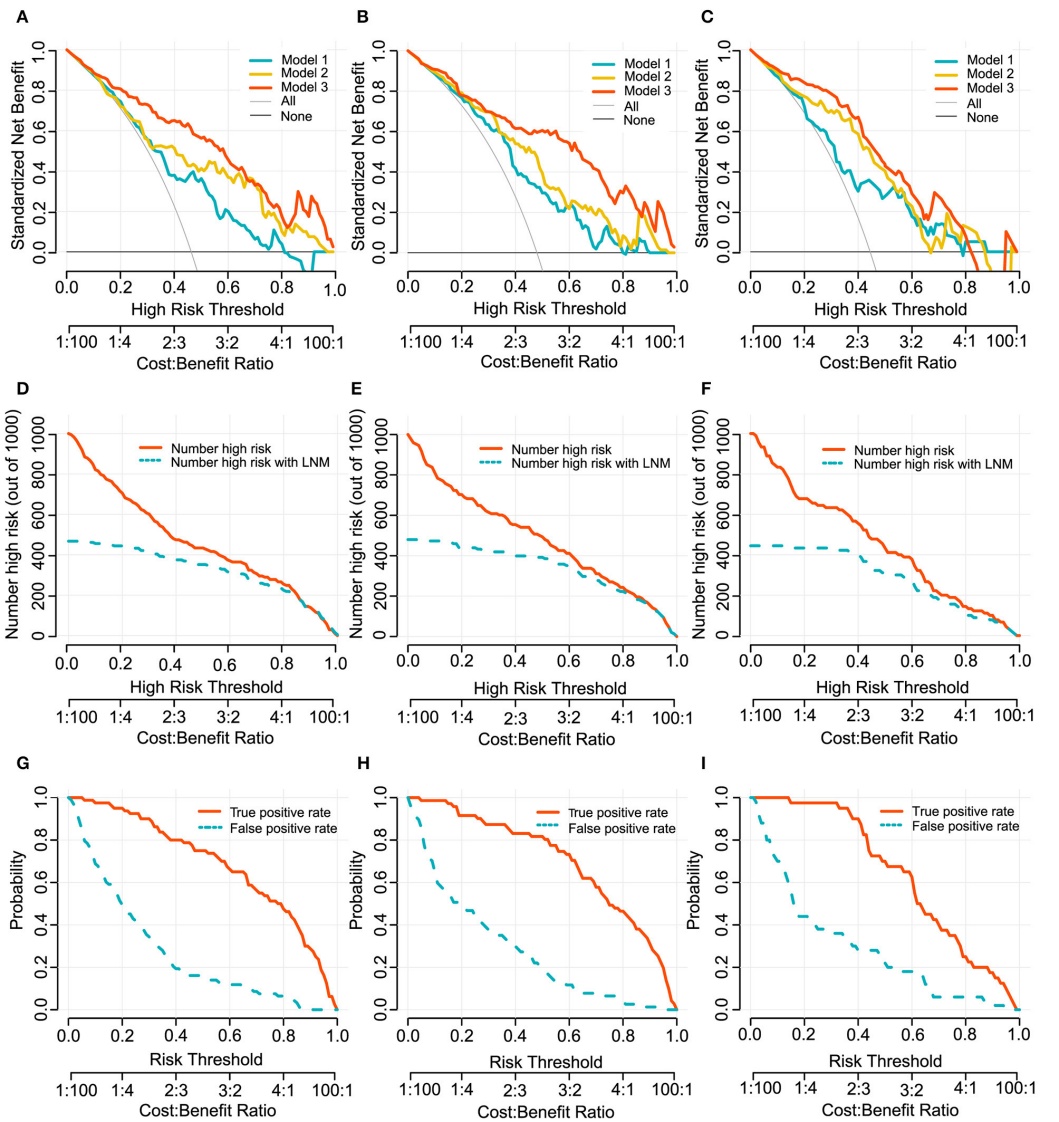
Decision curves of Model 1, 2, and 3 for predicting LN metastasis in development cohort **(A)**, internal validation cohort **(B)** and external validation cohort **(C)**. The x-axes and below line show the risk threshold and the cost-benefit ratio. The vertical axis shows the net benefit of standardization. The clinical impact curves for Model 3 shows in **(D–F)**. The red solid line shows the number of patients who would be regarded as high risk at the related risk threshold, and the blue dotted line indicates the true positive patients with LN metastasis. True- and false-positive rates with relate risk threshold were plotted in **(G–I)**. This figure contains similar information to a receiver operating characteristic curve, and also presents the true positive rate by a red solid line and false positive rate by a blue dotted line in each risk threshold. The first column **(A,D,G)**: development cohort. The second column **(B,E,H)**: internal validation cohort. The third column **(C,F,I)**: external validation cohort.

To provide clinicians with an easy-to-use tool, the radiomics nomogram was developed by Model 3 (Figure 3A). DCA plots (Figures 4A–C) of Model 3 showed that patients could get net benefit from the prediction model at the range of risk threshold from 0.3 to 0.8. And then, the clinical impact plot (Figures 4D–F) showed that, to illustrate at risk threshold of 0.5, of the 1,000 patients predicted, ~434, 493, and 433 were considered to have a high risk of developing LN metastases, of which ~326, 370, and 325 were true LN metastases in development, internal validation, and external validation cohort, respectively. Furthermore, information similar to the receiver operating characteristic curve (ROC) was presented by ROC components plot (Figures 4G–I), and the risk threshold corresponding to each true- and false-positive rate was clearly reflected.

## Discussion

In the present multicenter study, we developed and validated three predictive models for LN metastasis in patients with ESCC, including Model 1 (CT-reported LN status plus handcrafted-radiomics signature), Model 2 (Model 1 plus CV-radiomics signature), and Model 3 (Model 2 plus deep-radiomics signature). Our result showed that Model 3 outperformed the other two models in discrimination, calibration and clinical usefulness abilities, indicating that the addition of CV features and deep features into the predictive model can improve the prediction ability of LN metastasis in patients with ESCC.

Currently in clinical practice, preoperative assessment of LN metastasis in patients with ESCC is primarily diagnosed by radiologists based on radiological methods using LN size criteria, such as CT images. In our study, CT-reported LN status showed unsatisfactory discrimination (C-statistic, 0.655, in external validation cohort). This result was consistent with several previous reports (7, 32), indicating that the traditional size criteria cannot accurately reflect the metastatic status of LN, which leads to the insufficiency of CT diagnosis.

Many studies have suggested that medical images quantitative features could decode the biological characteristics of tumors at the genetic and cellular levels, which potentially improve tumor precision prediction and prognosis (10, 33, 34). We quantified CT images to biomedical features by different methods and select key image features to build radiomics signatures. Model 1 was developed with CT-reported LN status and handcrafted-radiomics signature, showing the discrimination with C-statistic of 0.728 in external validation cohort. In recent studies, Tan et al. (27) and Shen et al. (35) also developed a similar radiomics nomogram, which presented an AUC of 0.773 and 0.771 in the validation cohort, respectively. Although the effect of their handcrafted radiomics model was superior to Model 1 of our research, they did not have external validation. Moreover, we included more patients from different institutions and from different CT facilities while the same CT scanner was selected in Tan's study. Different CT image acquisitions made the difference in the radiomics features (36, 37), which might lead to bias and could explain the poor performance in Model 1.

When CV-radiomics signature and deep-radiomics signature were added to CT-reported and handcrafted-radiomics signature, the Model 3 showed a preferable discrimination in three cohorts. One of the reasons is that local features of computer vision excel in low computational complexity, no pre-learning process, no additional parameters to learn and highly robust to noise. The previous work also pointed out that local features based computer vision have the potential to provide relevant candidate diagnosis results for radiologists (38). This indicates that maybe computer vision can make full use of texture, shape, contour information to quantify heterogeneity of tumor. The other reason is, in contrast with predefined handcrafted features, deep radiomics features in the fine tuning model learn directly from image patches in a data-driven way and could provide supplement information to improve the performance of the model. Previous study showed that deep features extracted from the CT image combined with traditional features had potentially improve survival prediction ability in patients with lung cancer. In brief, CV-radiomics signature and deep-radiomics signature may be able to obtain more detailed information about tumor that cannot be mathematically defined.

To explore the incremental predictive value of CV- and deep-radiomics signature, we added them orderly to Model 1. The addition of a CV-radiomics signature to Model 1 significantly improved the reclassification performance in all cohorts. The updated Model 3, with the deep radiomics signature, further improved the reclassification performance (external validation cohort: NRI = 0.790; *p* < 0.001). As expected, the outperformance of Model 3 indicated that CV- and deep-radiomics features may provide more information and add predictive value for preoperative prediction of LN status of patients with ESCC. Our finding may also support that using a combination of signatures covering different aspects could be a promising approach to help improve precision medicine. Comparing with previous studies of handcrafted radiomics model (9, 27, 35), CV- and deep-radiomics features were added as independent signatures in our work, which significantly improved the model's predictive ability for LN metastasis of ESCC (C-statistic, 0.840, in external validation cohort).

Considering that evaluation methods (discrimination and calibration) of model performance could not reflect clinical relevance well, we applied DCA method to evaluate model clinical usefulness ability in the range of threshold probability in order to help make clinical decision preferably (39). In this study, the decision curve showed that if the risk threshold ranged from 0.3 to 0.8, Model 3 would add more benefit to predicting LN metastasis than the other models, and it may be supported as a potentially useful tool to help treatment decision making in clinical.

Some limitations were included in the study. Firstly, we used the limited population for analysis, which was especially not enough for deep learning study. Secondly, we used 2D features extracted from the maximum tumor instead of 3D features. Though 3D features which take the whole tumor into consideration may provide more information, previous studied mentioned that there was no significant improvement from 3D features comparing with 2D features (40, 41). The reason might be that 3D features were more sensitive to the variance of such as slice thickness and convolution kernel (42). However, the situation that images from different scanners is difficult to avoid in multicenter studies and retrospective studies. Accordingly, further studies are needed to find solutions for this problem and to further improve discrimination accuracy and generalization ability. Finally, previous studies have shown that gene events such as ZNF750 mutations were associated with metastasis in patients with ESCC (43). In future when genetic data is available, adding these gene markers may further improve model predictive value.

In conclusion, this study added computer vision radiomics signature and deep radiomics signatures in developing a multiple level CT radiomics preoperative prediction model for LN metastasis of patients with ESCC, which showed best prediction performance and clinical usefulness among the tested models. Our prediction model might be useful for identifying individual risk of LN metastasis and guiding personalize treatment.

## Data Availability Statement

The datasets generated for this study are available on request to the corresponding author.

## Ethics Statement

This multicenter retrospective study was approved by the Institutional Ethics Committee of two participating hospitals (Guangdong Provincial People's hospital, denote as Hospital 1; The Sixth Affiliated Hospital, Sun Yat-Sen University, denote as Hospital 2). Requirement for informed consent was waived.

## Author Contributions

CL, ZL, and ZZ: study conception and design. LW, XY, WC, and WL: data collection. LW, WC, and XY: data analysis and interpretation. LW and XY: manuscript writing. ZL, CL, WY, KZ, and XC: manuscript revise. All authors: manuscript review and final approval of manuscript.

### Conflict of Interest

The authors declare that the research was conducted in the absence of any commercial or financial relationships that could be construed as a potential conflict of interest.
