# Supplementary material for: Multiple Level CT Radiomics Features Preoperatively Predict Lymph Node Metastasis in Esophageal Cancer: A Multicentre Retrospective Study
Source: Front Oncol. 2020 Jan 21;9:1548. doi: 10.3389/fonc.2019.01548 (PMC6985546; doi:10.3389/fonc.2019.01548)
Supplement: Supplementary file 1 [file Table_1.DOCX]

***Supplementary material***

1. **Supplementary Data**
2. **Supplementary Methods**

S1. Handcrafted features extraction

S2. Computer vision features

S3. Deep learning features

S4. Signature construction

S5. Statistical analysis

1. **Supplementary Figures and Tables**

**3.1 Supplementary figures**

Figure S1: Discrimination box plots

**3.2 Supplementary Tables**

Table S1. Handcrafted- , CV- and deep- radiomics signature scores in three cohorts.

Table S2: Performance comparison of the LN metastasis prediction model by net reclassification improvement (NRI).

**1. Supplementary Data**

**Images Acquisition parameters**

The patients in this study were registered from two centers. All patients have underwent a contrast enhanced computed tomography (CT) scan from the neck to the abdomen. Detail description of the images acquisition parameters were presented as follows.

***(1). For the Guangdong Provincial People’s Hospital data*,** all CT scans were performed on three model machine: GE lightSpeed VCT, GE lightSpeed Ultra and Phillips iCT 256, parameters include:

1. Kilovolt peak (KVP): 120 kV
2. Effective mAs: 150 mAs
3. Rotation Time: 0.4s-0.6 s
4. Detector Collimation: 64 × 0.625 mm
5. Reconstruction Slice Thickness: 5.00 mm
6. Field of View: 350 × 350 mm^2^ (matrix: 512 × 512 pixels)

After routine non-enhanced CT, 1.5 ml/kg of iodinated contrast material (Ultravist 370, Bayer Schering Pharma) was injected into the vein at a rate of 3.0 to 3.5 mL/s via a pump syringe (Ulrich CT Plus). After 20s to 40s, the arterial phase contrast enhanced CT was implemented from neck to the abdomen.

***(2). For the Sixth Affiliated Hospital, Sun Yat-Sen University data***, all CT scans were performed on two model machine: GE Optima CT660, TOSHIBA Aquilion ONE, parameters include:

1. Kilovolt peak (KVP): 120 kV
2. Effective mAs: Auto mA
3. Rotation Time: 0.5s or 0.6s
4. Detector Collimation: 64 × 0.625 mm or 80 × 0.5 mm
5. Reconstruction Slice Thickness: 1.25 mm or 1 mm
6. Field of View: 250 × 250 mm^2^ or 220 × 220 mm^2^ (matrix: 512 × 512 pixels)

After routine non-enhanced CT, 1 to 1.5 ml/kg of the contrast agent iopromide 370 was injected into the vein at a rate of 2.5 to 4 ml/s by using the BAB injection method. After 20s to 30s, the arterial phase contrast enhanced CT was implemented from neck to the abdomen.

**2. Supplementary Methods**

**S1. Handcrafted features extraction**

3472 handcrafted features were extracted from each patient, which can be classified into four categories as follows:

1. Shape- and size-based features
2. First order features
3. Textural features
4. Wavelet features

**Category 1: Shape- and size-based features**

In this group of features, we have listed the feature formula of the two-dimensional shape- and size- of the tumor region. Let in the following definitions: *A* represents the area and *R* the radius of of circular with the same area as the tumor. We determined the following shape- and size- based features:

1. **Area**

*Area* = ${Num}_{pixels}d_{ps}^{2}$

Where $d_{ps}$ represents pixel space

1. **Circumference**

*Circumference* = $B_{pixels} d_{ps}$

Where $B_{pixels}$ represents the number of pixels of the boundary of ROI

1. **Max 2d diameter:** the max two-dimensional tumor diameter is defined as the largest pairwise Euclidean distance between the pixels on the circumference of the tumor boundary.
2. **Sphericity**

$$sphericity=\frac{2\sqrt{\pi*Area}}{Circumference}$$

3. **Circumference to area ratio** **($\boldsymbol{R}_{\boldsymbol{C/A}}$)**

$$R_{C/A}=\frac{Circumference}{Area}$$

Where *C* is circumference and *A* is area

1. **Circular disproportion (CD)**

$$CD=\frac{Circumference}{2\pi R}$$

2. **Roundness**

$$roundness=\frac{4\pi*Area}{{Circumference}^{2}}$$

**Category 2: First order features**

The following 14 statistical features were extracted.

Let **X** be the three dimensional image matrix with *N* voxels of the ROI and P be the first order histogram distribution with *N_g_* discrete intensity levels.

1. **Energy**

*Energy* = $\sum_{i}^{N} X(i)^{2}$

1. **Entropy**

*Entropy* = $\sum_{i=1}^{N_{g}} P(i)\log_{2}P(i)$

1. **Max Intensity:** The maximum intensity value of **X.**
2. **Min Intensity:** The minimum intensity value of **X**.
3. **Median:** The median intensity value of **X**.
4. **Mean**

*Mean* **=** $\frac{1}{N}\sum_{i}^{N} X(i)$

1. **Kurtosis**

*Kurtosis* = $\sqrt{\sum_{j=1}^{N_{g}} P(\mathrm{j})*(\mathrm{j}-\sum_{i=1}^{N_{g}} P(\mathrm{i})*\mathrm{i})^{4}}$

1. **Skewness**

*Skewness* = $\sqrt{\sum_{j=1}^{N_{g}} P(\mathrm{j})*(\mathrm{j}-\sum_{i=1}^{N_{g}} P(\mathrm{i})*\mathrm{i})^{3}}$

1. **Variance**

*Variance* = $\sqrt{\sum_{j=1}^{N_{g}} P(\mathrm{j})*(\mathrm{j}-\sum_{i=1}^{N_{g}} P(\mathrm{i})*\mathrm{i})^{2}}$

1. **Uniformity**

*Uniformity* = $\sum_{i=1}^{N_{g}} P(i)^{2}$

1. **Mean absolute deviation:** The mean of the absolute deviations of all pixels intensities around the mean intensity value
2. **Range:** The range of intensity values of **X.**
3. **Root mean square (*RMS*)**

*RMS =*$\sqrt{\frac{\sum_{i}^{N}X(i)^{2}}{N}}$

1. **Standard deviation (*SD*)**

$$SD=\sqrt{\frac{1}{N_{p}}\sum_{i=1}^{N_{p}} {(\mathbf{X}\left( i \right)\mathbf{-}\bar{X})}^{2}}$$

**Category 3: Textural features**

As the second order statistic texture features, texture features were calculated from the Gray Level Co-occurrence Matrix (GLCM), Gray Level Run Length Matrix (GLRLM), Gray level dependence matrix (GLDM), Gray Level Size Zone Matrix (GLSZM), and Neighborhood Gray Tone Difference Matrix (NGTDM).

***Gray-Level Co-Occurrence Matrix based features (GLCM)***

In GLCM, we defined as a matrix *M* (*i, j;*$\gamma$*,*$\alpha$) to indicate the relative frequency with intensity values of pixels (*i* and *j*) at the distance of $\gamma$ in direction $\alpha$.

Let:

*M*(*i, j*) be the co-occurrence matrix for an arbitrary*γ*and*α*, set*γ=1 andα=0*

*N_g_* be the number of discrete intensity levels in the images, set *N_g_* = 25,

*μ* be the mean of *M*(*i, j*),

$m_{x}(\mathrm{i})=\sum_{j=1}^{N_{g}} M(\mathrm{i},\mathrm{j})$be the marginal row probabilities,

$m_{y}(\mathrm{i})=\sum_{i=1}^{N_{g}} M(\mathrm{i},\mathrm{j})$be the marginal column probabilities, and *u_y_ ,μ_x_,* be the mean of *m_x_* .and *m_y_*

$HX=-\sum_{i=1}^{N_{g}} m_{x}(i)log(m_{x}(i)$,

$HY=-\sum_{i=1}^{N_{g}} m_{y}(i)log(m_{y}(i)$,

$HXY=-\sum_{i=1}^{N_{g}} \sum_{j=1}^{N_{g}} m(i,j)log(m(i,j))$,

$HXY1=-\sum_{i=1}^{N_{g}} \sum_{j=1}^{N_{g}} m(i,j)log(m_{x}(i)m_{y}(j))$.

$HXY2=-\sum_{i=1}^{N_{g}} \sum_{j=1}^{N_{g}} m_{x}(i)m_{y}(j)log(m_{x}(i)m_{y}(j))$.

1. **Energy**

*Energy* = $\sum_{i=1}^{N_{g}} \sum_{j=1}^{N_{g}} [\mathrm{M}(\mathrm{i},\mathrm{j})]^{2}$

1. **Contrast**

*Contrast* = $\sum_{i=1}^{N_{g}} \sum_{j=1}^{N_{g}} (\mathrm{i}-\mathrm{j})^{2}*M(\mathrm{i},\mathrm{j})$

1. **Entropy**

*Entropy* = $\sum_{i=1}^{N_{g}} \sum_{j=1}^{N_{g}} M(\mathrm{i},\mathrm{j})*\log_{2}M(\mathrm{i},\mathrm{j})$

1. **Homogeneity 1**

*Homogeneity_1* = $\sum_{i=1}^{N_{g}} \sum_{j=1}^{N_{g}} \frac{M(\mathrm{i},\mathrm{j})}{1+|\mathrm{i}-\mathrm{j}|}$

1. **Homogeneity 2**

*Homogeneity_2* = $\sum_{i=1}^{N_{g}} \sum_{j=1}^{N_{g}} \frac{M(\mathrm{i},\mathrm{j})}{1+|\mathrm{i}-\mathrm{j}|^{2}}$

1. **Correlation**

*Correlation =* $\frac{\sum_{i=1}^{N_{g}} \sum_{j=1}^{N_{g}} ijM(\mathrm{i},\mathrm{j})-\mu_{i}(\mathrm{i})\mu_{j}(\mathrm{j})}{\sigma_{x}(\mathrm{i})\sigma_{y}(\mathrm{j})}$

1. **Variance**

*Variance =* $\sum_{i=1}^{N_{g}} \sum_{j=1}^{N_{g}} (\mathrm{i}-\mu)^{2}M(\mathrm{i},\mathrm{j})$

1. **Sum Average**

*Sum_average =* $\sum_{i=2}^{2N_{g}} iM_{x+y}(\mathrm{i})$

1. **Dissimilarity**

*Dissimilarity =* $\sum_{i=1}^{N_{g}} \sum_{j=1}^{N_{g}} |\mathrm{i}-\mathrm{j}|\mathrm{M}(\mathrm{i},\mathrm{j})$

1. **Inverse Difference Moment (*IDM*)**

*IDM =* $\sum_{i=1}^{N_{g}} \sum_{j=1}^{N_{g}} \frac{\mathrm{M}(\mathrm{i},\mathrm{j})}{1+(\frac{|\mathrm{i}-\mathrm{j}|^{2}}{N^{2}})}$

1. **Autocorrelation**

*Autocorrelation =* $\sum_{i=1}^{N_{g}} \sum_{j=1}^{N_{g}} ijM(\mathrm{i},\mathrm{j})$

1. **Cluster Prominence**

*Cluster_prominence =* $\sum_{i=1}^{N_{g}} \sum_{j=1}^{N_{g}} \left[i+j-u_{x}-u_{y}\right]^{4}M(\mathrm{i},\mathrm{j})$

1. **Cluster Shade**

*Cluster_shade =* $\sum_{i=1}^{N_{g}} \sum_{j=1}^{N_{g}} \left[i+j-u_{x}-u_{y}\right]^{3}M(\mathrm{i},\mathrm{j})$

1. **Cluster Tendency**

*Cluster_tendency =* $\sum_{i=1}^{N_{g}} \sum_{j=1}^{N_{g}} \left[i+j-u_{x}-u_{y}\right]^{2}M(\mathrm{i},\mathrm{j})$

1. **Difference Entropy**

*Difference_entropy =* $-\sum_{i=0}^{N_{g}-1} M_{x-y}(i)\log_{2}\left[M_{x-y}(i)\right]$

1. **Maximum Probability (*Max_Prob*)**

*Max_Prob =* $max\{M(i,j)\}$

1. **Sum variance**

*Sum_variance =* $\sum_{i=2}^{2N_{g}} (i-SE)^{2}M_{x+y}(i)$

1. **Informational measure of correlation 1 (*IMC1*):**

*IMC1 =* $\frac{HXY-HXY1}{\max\left\{HX-HY\right\}}$

1. **Informational measure of correlation 2 (IMC2):**

*IMC2* = $\sqrt{1-e^{-2(HXY2-HXY)}}$

1. **Inverse Difference Moment Normalized (****IDMN):**

*IDMN* = $\sum_{i=1}^{N_{g}} \sum_{j=1}^{N_{g}} \frac{M(i,j)}{1+(\frac{\left|i-j\right|^{2}}{N^{2}})}$

1. **Inverse Difference Normalized (IDN):**

*IDN* = $\sum_{i=1}^{N_{g}} \sum_{j=1}^{N_{g}} \frac{M(i,j)}{1+(\frac{\left|i-j\right|}{N^{2}})}$

1. **inverse variance**

*inverse_variance =* $\sum_{k=1}^{N_{g}-1} \frac{p_{x-y}(k)}{k^{2}}$

***Gray Level Run Length Matrix based features (GLRLM)***

GLRLM calculates the gray level runs, which were defined as *P*(*i, j; θ*) to indicate the number of times *j* and gray level *i* appear consecutively in the direction *θ*.

Let:

*P*(*i, j; θ*) be the run-length matrix *P* for a direction *θ*, set *θ=0,*

*N_g_* be the number of discrete intensity values,

*N_r_* be the number of different run lengths, and

*N_p_* be the number of pixels in the ROI.

1. **Short Run Emphasis (*SRE*)**

*SRE =* $\frac{\sum_{i=1}^{N_{g}} \sum_{j=1}^{N_{r}} [\frac{P(\mathrm{i},\mathrm{j};\theta)}{j^{2}}]}{\sum_{i=1}^{N_{g}} \sum_{j=1}^{N_{r}} P(\mathrm{i},\mathrm{j};\theta)}$

1. **Long Run Emphasis (*LRE*)**

*LRE =* $\frac{\sum_{i=1}^{N_{g}} \sum_{j=1}^{N_{r}} j^{2}P(\mathrm{i},\mathrm{j};\theta)}{\sum_{i=1}^{N_{g}} \sum_{j=1}^{N_{r}} P(\mathrm{i},\mathrm{j};\theta)}$

1. **Gray-Level Nonuniformity (*GLN*)**

*GLN =* $\frac{\sum_{i=1}^{N_{g}} [\sum_{j=1}^{N_{r}} P(\mathrm{i},\mathrm{j};\theta)]^{2}}{\sum_{i=1}^{N_{g}} \sum_{j=1}^{N_{r}} P(\mathrm{i},\mathrm{j};\theta)}$

1. **Run-Length Nonuniformity (*RLN*):**

*RLN =* $\frac{\sum_{j=1}^{N_{r}} [\sum_{i=1}^{N_{g}} P(\mathrm{i},\mathrm{j};\theta)]^{2}}{\sum_{i=1}^{N_{g}} \sum_{j=1}^{N_{r}} P(\mathrm{i},\mathrm{j};\theta)}$

1. **Run Percentage (*RP*):**

*RP =* $\sum_{i=1}^{N_{g}} \sum_{j=1}^{N_{r}} \frac{P(\mathrm{i},\mathrm{j};\theta)}{N_{p}}$

1. **Low Gray Level Run Emphasis (*LGLRE*):**

*LGLRE =* $\frac{\sum_{i=1}^{N_{g}} \sum_{j=1}^{N_{r}} [\frac{P(\mathrm{i},\mathrm{j};\theta)}{i^{2}}]}{\sum_{i=1}^{N_{g}} \sum_{j=1}^{N_{r}} P(\mathrm{i},\mathrm{j};\theta)}$

1. **High Gray Level Run Emphasis (*HGLRE*):**

*HGLRE =* $\frac{\sum_{i=1}^{N_{g}} \sum_{j=1}^{N_{r}} i^{2}P(\mathrm{i},\mathrm{j};\theta)}{\sum_{i=1}^{N_{g}} \sum_{j=1}^{N_{r}} P(\mathrm{i},\mathrm{j};\theta)}$

1. **Short Run Low Gray Level Emphasis (*SRLGLE*):**

*SRLGLE =* $\frac{\sum_{i=1}^{N_{g}} \sum_{j=1}^{N_{r}} \frac{P(\mathrm{i},\mathrm{j};\theta)}{i^{2}j^{2}}}{\sum_{i=1}^{N_{g}} \sum_{j=1}^{N_{r}} P(\mathrm{i},\mathrm{j};\theta)}$

1. **Short Run High Gray Level Emphasis (*SRHGLE*):**

*SRHGLE =* $\frac{\sum_{i=1}^{N_{g}} \sum_{j=1}^{N_{r}} \frac{i^{2}P(\mathrm{i},\mathrm{j};\theta)}{j^{2}}}{\sum_{i=1}^{N_{g}} \sum_{j=1}^{N_{r}} P(\mathrm{i},\mathrm{j};\theta)}$

1. **Long Run Low Gray Level Emphasis (*LRLGLE*):**

*LRLGLE =* $\frac{\sum_{i=1}^{N_{g}} \sum_{j=1}^{N_{r}} \frac{j^{2}P(\mathrm{i},\mathrm{j};\theta)}{i^{2}}}{\sum_{i=1}^{N_{g}} \sum_{j=1}^{N_{r}} P(\mathrm{i},\mathrm{j};\theta)}$

1. **Long Run High Gray Level Emphasis (*LRHGLE*):**

*LRHGE =* $\frac{\sum_{i=1}^{N_{g}} \sum_{j=1}^{N_{r}} i^{2}j^{2}P(\mathrm{i},\mathrm{j};\theta)}{\sum_{i=1}^{N_{g}} \sum_{j=1}^{N_{r}} P(\mathrm{i},\mathrm{j};\theta)}$

***Gray level dependence matrix based features (GLDM)***

GLDM calculates gray level dependencies in an image, which were defined as *P(i,j)* represents the number of pixels containing *j* correlated gradations in the neighborhood of all pixelswhose gradation is *i.* Let:

*N_g_* be the number of discreet intensity values in the image

*N_d_* be the number of discreet dependency sizes in the image

*N_z_* be the number of dependency zones in the image, which is equal to $\sum_{i=1}^{N_{g}} \sum_{j=1}^{N_{d}} P(i,j)$

**P**(*i,j*) be the dependence matrix

*p*(*i,j*) be the normalized dependence matrix, defined as *p(i,j)* = $\frac{P(i,j)}{N_{z}}$

1. **Small Dependence Emphasis (*SDE*)**

*SDE=* $\frac{\sum_{i=1}^{N_{g}} \sum_{j=1}^{N_{d}} \frac{P(i,j)}{i^{2}}}{N_{z}}$

1. **Large Dependence Emphasis (*LDE*)**

*LDE* = $\frac{\sum_{i=1}^{N_{g}} \sum_{j=1}^{N_{d}} P(i,j)j^{2}}{N_{z}}$

1. **Gray Level Non-Uniformity (*GLN*)**

*GLN* = $\frac{\sum_{i=1}^{N_{g}} (\sum_{j=1}^{N_{d}} P(i,j))^{2}}{N_{z}}$

1. **Dependence Non-Uniformity (*DN*)**

*DN* = $\frac{\sum_{j=1}^{N_{d}}(\sum_{i=1}^{Ng} P(i,j))^{2}}{N_{z}}$

1. **Dependence Non-Uniformity Normalized (*DNN*)**

*DNN* = $\frac{\sum_{j=1}^{N_{d}} (\sum_{i=1}^{Ng}P(i,j))^{2}}{N_{z}^{2}}$

1. **Dependence Entropy (*DE*)**

*DE* = $\sum_{i=1}^{N_{g}} \sum_{j=1}^{N_{d}} p(i,j)\log_{2}(p(i,j)+\varepsilon)$

1. **Low Gray Level Emphasis (*LGLE*)**

*LGLE* = $\frac{\sum_{i=1}^{N_{g}} \sum_{j=1}^{N_{d}} \frac{P(i,j)}{i^{2}}}{N_{z}}$

1. **High Gray Level Emphasis (*HGLE*)**

*HGLE* =

1. **Small Dependence Low Gray Level Emphasis (*SDLGLE*)**

*SDLGLE* =

1. **Small Dependence High Gray Level Emphasis (*SDHGLE*)**

*SDHGLE* =

1. **Large Dependence Low Gray Level Emphasis (*****LDLGLE*)**

*LDLGLE* = $\frac{\sum_{i=1}^{N_{g}} \sum_{j=1}^{N_{d}} \frac{P(i,j)j^{2}}{i^{2}}}{N_{z}}$

1. **Large Dependence High Gray Level Emphasis (*****LDHGLE*)**

*LDHGLE* =

***Gray Level Size Zone Matrix based features (GLSZM)***

GLSZM based features were high-order statistical texture features, which were defined as *P*(*i, j*) to indicate the areas of size j and gray level i.

Let:

*P*(*i, j*) be the size zone of matrix *P*,

*N_g_* be the number of discrete intensity values,

*N_r_* be the number of different areas sizes,

*N_p_* be the number of pixels in the ROI.

1. **Small** **Area Emphasis (*SAE*):**

*SAE =* $\frac{\sum_{i=1}^{N_{g}} \sum_{j=1}^{N_{r}} [\frac{P(\mathrm{i},\mathrm{j})}{j^{2}}]}{\sum_{i=1}^{N_{g}} \sum_{j=1}^{N_{r}} P(\mathrm{i},\mathrm{j})}$

1. **Large Area Emphasis (*LAE*):**

*LAE =* $\frac{\sum_{i=1}^{N_{g}} \sum_{j=1}^{N_{r}} j^{2}P(\mathrm{i},\mathrm{j})}{\sum_{i=1}^{N_{g}} \sum_{j=1}^{N_{r}} P(\mathrm{i},\mathrm{j})}$

1. **Gray Level Nonuniformity (*GLN*):**

*GLN =* $\frac{\sum_{i=1}^{N_{g}} [\sum_{j=1}^{N_{r}} P(\mathrm{i},\mathrm{j})]^{2}}{\sum_{i=1}^{N_{g}} \sum_{j=1}^{N_{r}} P(\mathrm{i},\mathrm{j})}$

1. **Gray level non-uniformity normalized (*GLNN*)**

*GLNN* =

1. **Size Zone Non-uniformity (*SZN*):**

*SZN =* $\frac{\sum_{j=1}^{N_{r}} [\sum_{i=1}^{N_{g}} P(\mathrm{i},\mathrm{j})]^{2}}{\sum_{i=1}^{N_{g}} \sum_{j=1}^{N_{r}} P(\mathrm{i},\mathrm{j})}$

1. **Size Zone Non-uniformity normalized (*SZNN*):**

*SZNN =*

1. **Zone Percentage (*ZP*):**

*ZP =* $\sum_{i=1}^{N_{g}} \sum_{j=1}^{N_{r}} \frac{P(\mathrm{i},\mathrm{j})}{N_{p}}$

1. **Low Gray Level Zone Emphasis (*LGLZE*):**

*LGLZE =* $\frac{\sum_{i=1}^{N_{g}} \sum_{j=1}^{N_{r}} [\frac{P(\mathrm{i},\mathrm{j})}{i^{2}}]}{\sum_{i=1}^{N_{g}} \sum_{j=1}^{N_{r}} P(\mathrm{i},\mathrm{j})}$

1. **High Gray Level Zone Emphasis (*HGLZE*):**

*HGLZE =* $\frac{\sum_{i=1}^{N_{g}} \sum_{j=1}^{N_{r}} i^{2}P(\mathrm{i},\mathrm{j})}{\sum_{i=1}^{N_{g}} \sum_{j=1}^{N_{r}} P(\mathrm{i},\mathrm{j})}$

1. **Small Area Low Gray-Level Emphasis (*SALGLE*):**

*SALGLE =* $\frac{\sum_{i=1}^{N_{g}} \sum_{j=1}^{N_{r}} \frac{P(\mathrm{i},\mathrm{j})}{i^{2}j^{2}}}{\sum_{i=1}^{N_{g}} \sum_{j=1}^{N_{r}} P(\mathrm{i},\mathrm{j})}$

1. **Small Area High Gray-Level Emphasis (*SAHGLE*):**

*SAHGLE =* $\frac{\sum_{i=1}^{N_{g}} \sum_{j=1}^{N_{r}} \frac{i^{2}P(\mathrm{i},\mathrm{j})}{j^{2}}}{\sum_{i=1}^{N_{g}} \sum_{j=1}^{N_{r}} P(\mathrm{i},\mathrm{j})}$

1. **Large Area Low Gray-Level Emphasis (*LALGLE*):**

*LALGLE =* $\frac{\sum_{i=1}^{N_{g}} \sum_{j=1}^{N_{r}} \frac{j^{2}P(\mathrm{i},\mathrm{j})}{i^{2}}}{\sum_{i=1}^{N_{g}} \sum_{j=1}^{N_{r}} P(\mathrm{i},\mathrm{j})}$

1. **Large Area High Gray-Level Emphasis (*LAHGLE*):**

*LAHGLE =* $\frac{\sum_{i=1}^{N_{g}} \sum_{j=1}^{N_{r}} i^{2}j^{2}P(\mathrm{i},\mathrm{j})}{\sum_{i=1}^{N_{g}} \sum_{j=1}^{N_{r}} P(\mathrm{i},\mathrm{j})}$

***Neighborhood Gray Tone Difference Matrix based features (NGTDM)***

NGTDM calculates the difference between a gray value and the average gray value of its neighbours within a distance. which were defined as *S(i)* to indicate the sum of the absolute value between gray intensity level i and it’s neighbors’ average intensity.

Let:

*S(i)* be the sum of absolute value between gray intensity level i and its neighbors’ average intensity,

*C(i)* be the number of pixels with the gray intensity level I,

*N_g_* be the number of discrete intensity values.

1. **Coarseness:**

*Coarseness* = $\frac{1}{\varepsilon+\sum_{i=1}^{N_{g}} \frac{C(\mathrm{i})\mathrm{S}(\mathrm{i})}{\sum_{i=1}^{N_{g}} C(\mathrm{i})}}$

1. **Contrast:**

*Contrast* = $\frac{\sum_{i=1}^{N_{g}} \sum_{j=1}^{N_{g}} C(\mathrm{i})\mathrm{C}(\mathrm{j})(\mathrm{i}-\mathrm{j})^{2}}{(\sum_{i=1}^{N_{g}} C(\mathrm{i}))^{2}}*\sum_{i=1}^{N_{g}} S(\mathrm{i})*\frac{1}{N_{g}(\mathrm{N}_{g}-1)\sum_{i=1}^{N_{g}} C(\mathrm{i})}$

1. **Busyness:**

*Busyness =*$\frac{\sum_{i=1}^{N_{g}} C(\mathrm{i})\mathrm{S}(\mathrm{i})}{\sum_{i=1}^{N_{g}} \sum_{j=1}^{N_{g}} (\mathrm{iC}(\mathrm{i})-\mathrm{jC}(\mathrm{j}))}, C(\mathrm{i})\neq0,\mathrm{C}(\mathrm{j})\neq0$

1. **Complexity:**

*Complexity* = $\frac{1}{(\sum_{i=1}^{N_{g}} C(\mathrm{i}))^{2}}*\sum_{i=1}^{N_{g}} \sum_{j=1}^{N_{g}} \frac{|\mathrm{i}-\mathrm{j}|(\mathrm{C}(\mathrm{i})\mathrm{S}(\mathrm{i})+\mathrm{C}(\mathrm{j})\mathrm{S}(\mathrm{j}))}{(\mathrm{C}(\mathrm{i})+\mathrm{C}(\mathrm{j}))}, C(\mathrm{i})\neq0,\mathrm{C}(\mathrm{j})\neq0$

1. **Strength:**

*Strength* = $\frac{\sum_{i=1}^{N_{g}} \sum_{j=1}^{N_{g}} (\mathrm{C}(\mathrm{i})-\mathrm{C}(\mathrm{j}))*(\mathrm{i}-\mathrm{j})^{2}}{\sum_{i=1}^{N_{g}} C(\mathrm{i})*\sum_{i=1}^{N_{g}} S(\mathrm{i})}, C(\mathrm{i})\neq0,\mathrm{C}(\mathrm{j})\neq0$

**Category 4: Wavelet features: first order statistical and texture features of a wavelet filtered image.**

1) With the Laplacian of Gaussian filter, four filter parameters applied: 1.0, 1.5, 2.0, 2.5. The laplacian of Gaussian filter ($\nabla^{2}G$) distribution is defined as follows:

$$\nabla^{2}G\left( x,y \right)=\frac{-1}{\pi\sigma^{4}}(1-\frac{x^{2}+y^{2}}{2\sigma^{2}})exp(-\frac{x^{2}+y^{2}}{2\sigma^{2}})$$

2) With the wavelet, we used ten different wavelet functions to filter an original image. X_LL_, X_LH_, X_HL_, X_HH_ are intensity value of the transformation images from original images by filters. L represents low pass filter and H for high pass filter. X_LL_ represents the intensity value from directional filtering of low pass filter on the x-direction, and low pass filter on the y-direction. In this study: db1, db10, sym8, coif3, bior2.6, bior3.9, bior4.4, rbio1.5, rbio3.3, rbio6.8 wavelet functions were applied.

**S2.** **Computer vision features**

Computer vision features (CVFs), mainly include local features and global features, are widely used for object recognition, image registration, image retrieval and texture classification. In this study, we used machine vision features to predict lymph node metastasis status in esophageal cancer. Hence, we choose four groups CVFs for analysis:

1. Speeded Up Robust Features (SURF). SURF is an image processing algorithm proposed by Herbert Bay et al in 2006 [1]. The SURF algorithm is applied to extract distinctive features by marking the intensity distribution of pixels near the point of interest (POI) in the region of interest (ROI). We used this algorithm extracted 640 features for the prediction of LN metastasis.
2. Local Binary Pattern (LBP), a type of feature descriptor, was described by T.Ojala [2]. It has shown with significant advantages in texture classification. 66 features were included in this study.
3. Histogram of Oriented Gradients (HOG), a feature descriptor, which were mainly used in object detection in image processing, composing features by calculating and statistic the gradient direction histogram of the local region of the image. 324 features were calculated.
4. Haar-likes features, which are a category of digital image features that are widely used in object recognition. This feature mainly used to reflect the grayscale changes of the image.4096 features were calculated in this work.

***Group 1: Speeded Up Robust Features (SURF)***

SURF is an image processing algorithm proposed by Herbert Bay et al [1] in 2006. The SURF algorithm is applied to extract distinctive features by marking the intensity distribution of pixels near the point of interest (POI) in the region of interest (ROI). In this study, the POI in each ROI was positioned by a Hessian-Matrix based blob detector and choosed by the maximum determinant of the Hessian-Matrix in directions of x and y. The orientation of every POI was also calculated by a fixed radiu. Then, the POI was segmented into 4 × 4 square sub-regions, and each sub-region was filtered by Haar wavelet, the responses from the Haar wavelet were extracted at 5 × 5 particular region points. Since the number of POI points detected by surf on different images is different, for the convenience of analysis, we select the 10 strongest POIs for feature extraction. Finally, 640 surf features were extracted for each patients.

***Group 2: Local Binary Pattern (LBP)***

LBP feature is a gray-level descriptor used to describe the texture of images. The original LBP operator performs LBP encoding for each pixel in the image utilized the 3 × 3 neighborhoods pixels. Specifically, use the center pixel value as the threshold, and the surrounding pixels value are compared with this threshold. If the pixel value is more than the threshold, the pixel value was encoded to 1, otherwise, encoded to 0. And then, the binary values were converted to decimal represents the area. The process of LBP encoding is illustrated in Fig. 1.


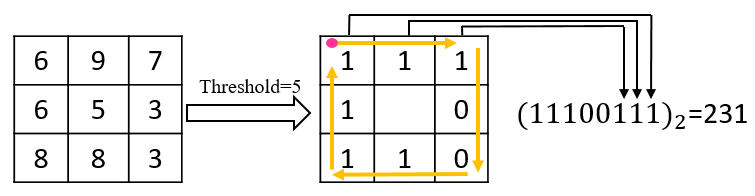


Fig. 1 The LBP encoding process illustration

The process can describe by the follow formula:

$${LBP}_{A,R}\left( X_{c},Y_{c} \right)=\sum_{A=0}^{A-1} S(P_{A}-P_{C})2^{A}$$

Where,$\left( X_{c},Y_{c} \right)$ is the pixel coordinates in the image, $P_{C}$ represents the center pixel value and $P_{A}$ represents neighborhoods pixel value. R is the radius used to determine neighborhoods. The function $S(x)$ defined as follows:

$$S\left( x \right)=\left\{ \begin{aligned} 0,x<0 \\ 1,x\geq0 \end{aligned} \right.$$

Ojala et al improved the classic LBP operator, introducing a rotation invariance and uniform pattern called uniform LBP. In other words, if the binary pattern includes at most two bitwise transitions from 0 to 1 or 1 to 0 when the bit pattern is traversed circularly, we called this local binary pattern as uniform LBP. A neighborhood of 64 sampling points on a circle of radius of 8 was used in this study.

When the cyclic binary number corresponding to an LBP has two hops from 0 to 1 or from 1 to 0, the binary corresponding to the LBP is called an equivalent mode class.

***Group 3: Histogram of Oriented Gradients (HOG)***

HOG, a feature descriptor algorithm, which was proposed by Dalal el ta [3], and mainly used in object recognition in image processing, composing features by calculating and statistic the gradient direction histogram of the local region of the image. Before executing the HOG algorithm, the image was fragmented into the smaller sub-regions (called cells), and the histogram of gradient orientations are calculated based on the pixels of the cells. All histograms of cells are then combined to form the image features. Primary steps for calculating HOG were presented as follows:

1) *Computation of the gradient.* The gradient value can be calculated by the formulas (Sobel operator was used):

$$\left| Gradient \right|=\sqrt{I_{x}^{2}+I_{y}^{2}}$$

$$\theta=arctan(\frac{I_{y}}{I_{x}})$$

where $I_{x}$ and $I_{y}$ are gradient values in the horizontal and vertical directions.

2) *Orientation binning.* This step is to create the cell histograms. HOG cells are circular and the channels of histogram has both signed (0-360 degrees) and unsigned (0-180 degrees). In this study, a bin of 9 (0, 20, 40, 60, 80, 100, 120, 140, 160) with unsigned histogram channel was adopt.

3) *Blocks construction and normalization.* Each block is composed of several cells. There are two kinds of geometrics for generating the block, which are circular HOG and rectangular HOG. For blocks normalization, L2-norm was applied and defined as follow:

$$\varphi=\frac{v}{\sqrt{\left\| v \right\|_{2}^{2}+e^{2}}}$$

Where *v* is a vector and *e* is a constant

In this study, the images were resized into 64×64, number of bins: 9, cell size: 16×16, each four cells form a block. Finally, 324 HOG features were extracted.

***Group 4: Haar-like Features (Haar)***

Haar-like feature has been widely used for object detection, which converts the original image into an integral image, and achieves fast calculations on regions of interest at different scales by utilizing integral images. Haar features are rectangular features, at each pixel, a rectangular region is defined, and different types of Haar features can be calculated (show in Fig. 2.).


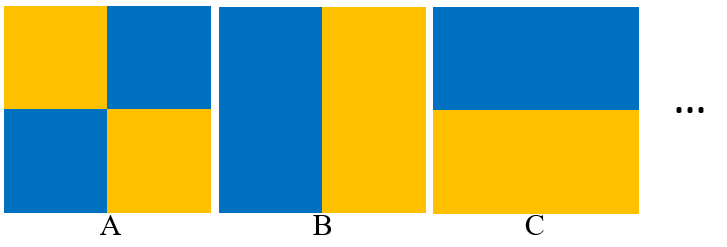


Fig.2 the types of Haar-like feature descriptors

Each Haar feature descriptor contains two connected rectangles: blue and yellow block. The Haar features could be computed by following formula:

$$g\left( x \right)=\sum_{B} B\_P-\sum_{Y} Y\_P$$

where, *B* represents a blue rectangle. *B_P* for pixel value of blue rectangle. *Y* represents a yellow rectangle. *Y_P* for pixel value of yellow rectangle.

To control the number of Haar-like features, we resized the image to 16×16 before extracting the feature. And the first type of Haar-like feature descriptors in fig2 was applied. Finally, 4096 Haar-like features were extracted.

***Computer vision features naming rule***

For the four types of features, they are named to ${surf}_{\alpha}$, ${lbp}_{\beta}$, ${hog}_{\gamma}$ and ${haar}_{\delta}$ respectively, where $\alpha\epsilon[1, 640]$, $\beta\epsilon[1, 66]$, $\gamma\epsilon[1, 324]$ and $\delta\epsilon[1, 4096]$.

**S3.** **Deep learning features**

***Images rescale***

CT image is single channel gray image, however, with a wider range of gray level (1×16 bits). In order to match the input of the pre-trained CNN-F model, three steps were performed. First, the largest tumor area slicer was selected from all slicers for each patient, and manually segmented the tumor area along the tumor boundary. Then, cropped the segmented tumor area and resized to 224×224 pixel2 by bicubic interpolation. Finally, the resized single channel image was encoded into a three channel image and allowed to input the model.

***Pre-training***

In this study, CNN-Fast (CNN-F) models was used as a feature extractor for deep learning features extraction. This model pre-trained on ILSVRC-2012 dataset and the training parameters are consistent with this study [4]: momentum 0.9, weight decay 5×10-4, initial learning rate 10-2, when the validation error stops decreasing, the initial rate drops to one tenth.

***Transfer learning***

Transfer learning is a technique that through transfer of knowledge learned from a previous task to a new related task. In this study, in order to overcome the small sample size, we use transfer learning to transfer the model pre-trained on the ILSVRC-2012 dataset to the esophageal cancer dataset to predict the metastatic state of the lymph nodes. As a feature extractor, the last fully connected layer of the model was removed.

***Deep features naming rule***

A total of 4096 features were extracted, we named the deep features as $V_{i}, i \epsilon[1,4096]$.

**S4. Signature construction**

***Handcrafted-radiomics signature***

5 key handcrafted features were selected to build a handcrafted radiomics signature by logistic regression after performing feature selection strategies. Handcrafted-radiomics score was computed by follow formula:

${Score}_{Handcrafted}$ = -0.196 +0.324$NGTDM\_Busyness_{1.0}^{LoG}$ -

0.345$GLSZM\_SALGLE_{X_{HL}}^{bior2.8}$ + 0.216$GLDM\_SDHGLE_{X_{LL}}^{coif4}$ +

0.168$GLCM\_Maximum\_probability_{X_{HL}}^{bior2.8}$ -0.401$Mean_{X_{HL}}^{bior2.8}$

***Computer vision radiomics signature***

7 key computer vision features were extracted after feature selection. A signature could be construct by logistic regression, and computer vision radiomics score can computed by follow formula:

$${Score}_{ComputerVision}=-0.418+0.415{haar}_{197}+{0.719haar}_{390}-{0.562haar}_{866}-{0.400lbp}_{47}-0.508{haar}_{315}-0.191{haar}_{2802}-0.436{hog}_{69}$$

***Deep radiomics signature***

After feature selection, 9 key deep features were selected to build a signature by using logistic regression. Deep radiomics score was computed by follow formula:

$${Score}_{Deep}=-0.332+0.343V_{142}-0.493V_{874}-0.704V_{3081}$$

$$-0.233V_{2160}+0.393V_{378}+0.413V_{1960}+0.542V_{704}$$

$$+0.265V_{618}+0.295V_{2884}$$

**S5. Statistical analysis**

R packages used in this study:

1. psych: intraclass correlation analysis
2. randomForest, caret: implement Forest-Recursive Feature Elimination (RF-RFE) algorithm
3. rms: nomograms and calibration plots
4. rmda: decision curve analysis and clinical impact plots
5. PredictABEL: calculate discrimination slope and plot discrimination box
6. ResourceSelection: Hosmer-Lemeshow test
7. Hmisc: for Net Reclassification Improvement (NRI)

**3. Supplementary Figures and Tables**

**3.1 Supplementary Figures**

1. Figure S1


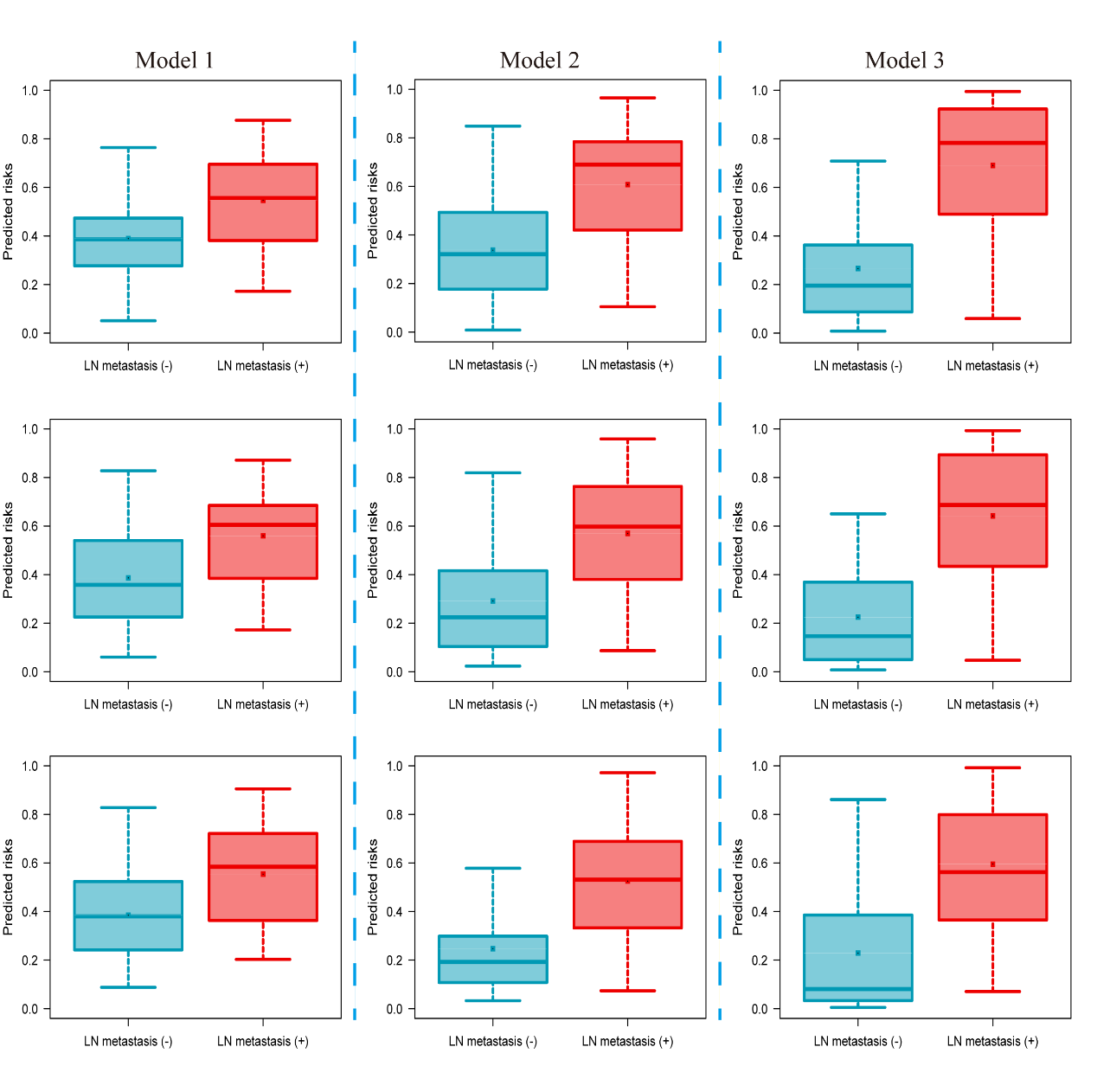


Figure S1. Box plots of predicted probabilities without (blue) and with (red) the LN metastasis

**3.2 Supplementary Tables**

**Table S1. Handcrafted- , CV- and deep- radiomics signature scores in three cohorts**

|  | Development Cohort | |  | Internal validation Cohort | |  | External validation Cohort | |  |
| --- | --- | --- | --- | --- | --- | --- | --- | --- | --- |
|  | LNM (-) | LNM (+) | p | LNM (-) | LNM (+) | p | LNM (-) | LNM (+) | p |
| H-Radscore | -0.223  (-0.697 0.123) | 0.007  (-0.362 0.313) | <0.001 | -0.251  (-0.629 0.073) | 0.016  (-0.201 0.247) | <0.001 | -0.328  (-0.781 0.133) | -0.016  (-0.293 0.3112) | 0.006 |
| CV-Radscore | -0.411  (-1.016 -0.013) | 0.148  (-0.326 0.638) | <0.001 | -0.798  (-1.396 -0.235) | -0.049  (-0.497 0.445) | <0.001 | -1.150  (-1.696 -0.533) | 0.072  (-1.167 0.562) | <0.001 |
| D-Radscore | -0.705  (-1.565 0.027) | 0.371  (-0.198 1.440) | <0.001 | -0.859  (-1.596 -0.146) | 0.282  (-0.443 1.142) | <0.001 | -0.764  (-1.796 -0.164) | 0.132  (-0.223 0.610) | <0.001 |

Note. Each radiomics score presents with media and interquartile ranges in parentheses.

H-Radscore: handcrafted radiomics score

CV-Radscore: computer vision radiomics score

D-Radscore: deep radiomics score

LNM (-): lymph node metastasis negative

LNM (+): lymph node metastasis positive

**Table S2.** **Performance comparison of the LN metastasis prediction model in patients with esophageal squamous cell carcinoma by net reclassification improvement (NRI)**

|  | Model Comparisons | NRI (95%) | p-value |
| --- | --- | --- | --- |
| Development cohort | Model 2 vs. Model 1 | 0.708 (0.429 – 0.988) | 6.64e-07 |
|  | Model 3 vs. Model 1 | 1.084 (0.834 – 1.334) | 1.79e-17 |
|  | Model 3 vs. Model 2 | 0.826 (0.555 – 1.098) | 2.46e-09 |
| Internal validation cohort | Model 2 vs. Model 1 | 0.709 (0.418 – 1.000) | 1.85e-06 |
|  | Model 3 vs. Model 1 | 0.874 (0.591 – 1.156) | 1.32e-09 |
|  | Model 3 vs. Model 2 | 0.722 (0.423 – 1.022) | 2.32e-06 |
| External validation cohort | Model 2 vs. Model 1 | 0.870 (0.508 – 1.232) | 2.44e-06 |
|  | Model 3 vs. Model 1 | 0.720 (0.335 – 1.105) | 2.47e-04 |
|  | Model 3 vs. Model 2 | 0.790 (0.408 – 1.172) | 5.14e-05 |

Note. NRI: net reclassification improvement

**References**

1. Bay, H., T. Tuytelaars, and L. Van Gool. *Surf: Speeded up robust features*. in *European conference on computer vision*. 2006. Springer.

2. Ojala, T., et al., *Multiresolution gray-scale and rotation invariant texture classification with local binary patterns.* 2002(7): p. 971-987.

3. Dalal, N. and B. Triggs. *Histograms of oriented gradients for human detection*. 2005.

4. Krizhevsky, A., I. Sutskever, and G.E. Hinton. *Imagenet classification with deep convolutional neural networks*. in *Advances in neural information processing systems*. 2012.
